# Supplementary material for: Who reported having a high-strain job, low-strain job, active job and passive job? The WIRUS Screening study
Source: PLoS One. 2019 Dec 30;14(12):e0227336. doi: 10.1371/journal.pone.0227336 (PMC6936855; doi:10.1371/journal.pone.0227336)
Supplement: S1 Appendix — nsNon-significant difference; *Significant difference (p < .05); an = 4487; bn = 18000, data obtained from included companies’ personell records; cn = 2800000, data obtained from Statistics Norway [44]; dn = 849620, data obtained from Statistics Norway [44]; eonly state sector employees, n = 159389, data obtained from Statistics Norway [45]; fPrimary/lower secondary; gUpper secondary; hUniversity/college; jDifferences tested with chi-square tests. (DOCX) [file pone.0227336.s001.docx]

**S1 Appendix. Study selection analyses (comparisons between study sample, invited sample, national workforce and public sector)**

| Distributions of gender, age and education (percentages) | | | | |
| --- | --- | --- | --- | --- |
| Variable | Study sample^a^ | Invited sample^b^ | National workforce^c^ | Public sector^d^ |
| Gender |  |  |  |  |
| Male | 33.1 | 34.8 | 52.7 | 30.2 |
| Female | 66.9 | 65.2 | 47.3 | 69.8 |
| Age |  |  |  |  |
| ≤39 | 32.1 | 36.3 | 45.0 | - |
| ≥40 | 67.9 | 63.7 | 55.0 | - |
| Education |  |  |  |  |
| Level 1^f^ | 2.8 | - | 16.3 | - |
| Level 2^g^ | 23.9 | - | 42.3 | - |
| Level 3^h^ | 73.3 | - | 41.4 | 72.7^e^ |
| Differences in percentage points and *p* values^j^ (gender, age and education) | | | | |
| Variable | | Invited sample^b^ | National workforce^c^ | Public sector^d^ |
| Gender (% males) | |  |  |  |
| Study sample^a^ | | 1.7 (.032)* | 19.6 (<.001)* | 2.9 (<.001)* |
| Age (% ≤39) | |  |  |  |
| Study sample^a^ | | 4.2 (<.001)* | 12.9 (<.001)* | - |
| Education (% level 3) | |  |  |  |
| Study sample^a^ | | - | 31.9 (<.001)* | 0.6 (.374)^ns^ |
